# MEK inhibitors: a promising targeted therapy for cardiovascular disease

**DOI:** 10.3389/fcvm.2024.1404253

**Published:** 2024-07-01

**Authors:** Khaled A. K. Mohammed, Paolo Madeddu, Elisa Avolio

**Affiliations:** ^1^ Bristol Heart Institute, Bristol Medical School, University of Bristol, Bristol, United Kingdom; ^2^ Department of Cardiothoracic Surgery, Faculty of Medicine, Assiut University, Assiut, Egypt

**Keywords:** aortic aneurysm, atherosclerosis, cardiac hypertrophy, cardiovascular disease, ERK, heart failure, MEK inhibitor, myocardial infarction

## Abstract

Cardiovascular disease (CVD) represents the leading cause of mortality and disability all over the world. Identifying new targeted therapeutic approaches has become a priority of biomedical research to improve patient outcomes and quality of life. The RAS-RAF-MEK (mitogen-activated protein kinase kinase)-ERK (extracellular signal-regulated kinase) pathway is gaining growing interest as a potential signaling cascade implicated in the pathogenesis of CVD. This pathway is pivotal in regulating cellular processes like proliferation, growth, migration, differentiation, and survival, which are vital in maintaining cardiovascular homeostasis. In addition, ERK signaling is involved in controlling angiogenesis, vascular tone, myocardial contractility, and oxidative stress. Dysregulation of this signaling cascade has been linked to cell dysfunction and vascular and cardiac pathological remodeling, which contribute to the onset and progression of CVD. Recent and ongoing research has provided insights into potential therapeutic interventions targeting the RAS-RAF-MEK-ERK pathway to improve cardiovascular pathologies. Preclinical studies have demonstrated the efficacy of targeted therapy with MEK inhibitors (MEKI) in attenuating ERK activation and mitigating CVD progression in animal models. In this article, we first describe how ERK signaling contributes to preserving cardiovascular health. We then summarize current knowledge of the roles played by ERK in the development and progression of cardiac and vascular disorders, including atherosclerosis, myocardial infarction, cardiac hypertrophy, heart failure, and aortic aneurysm. We finally report novel therapeutic strategies for these CVDs encompassing MEKI and discuss advantages, challenges, and future developments for MEKI therapeutics.

## 1 Introduction

Cardiovascular disease (CVD) includes a heterogeneous group of health conditions that affect the heart and blood vessels. It is the leading cause of morbidity and mortality worldwide, imposing a substantial burden on healthcare systems (1–3). CVD encompasses a wide range of cardiac and arterial disorders, including myocardial infarction (MI), atherosclerosis, cardiac hypertrophy, heart failure, and aortic aneurysm, among others (3). What unites these conditions is their joint impact on the heart and circulatory system, often resulting in compromised blood flow and potentially life-threatening consequences (3).

The multifaceted nature of CVD requires a holistic approach to prevention, diagnosis, and treatment. CVD risk factors are numerous and can be categorized into modifiable and non-modifiable (4). Modifiable risk factors include lifestyle choices such as unhealthy diet, physical inactivity, smoking, and excessive alcohol consumption (5). Non-modifiable factors encompass genetic predisposition, family history, age, and gender (6). With the global aging population and an increasing prevalence of lifestyle-related risk factors, CVD is projected to remain a significant public health concern (7–9). Researchers, clinicians, and healthcare professionals continue to work jointly to advance understanding of these pathologies, develop innovative treatments, and improve preventive strategies to reduce the global impact of CVD (10).

Targeted therapies have emerged as a pivotal and transformative approach to managing CVD, representing a paradigm shift in how we diagnose, treat, and prevent these conditions (11, 12). The significance of targeted therapies in CVD reflects a growing understanding of the complex molecular and genetic factors underlying these disorders (1, 13–15). This approach offers the potential for more effective and personalized treatments and improved patient outcomes (11). The primary advantage of targeted therapies is their precision in addressing the root causes of the diseases (16). Instead of relying solely on broad-spectrum medications, these therapies can pinpoint specific molecular pathways, proteins, and cellular processes involved in disease pathogenesis (17). Targeted therapies could halt or even reverse disease progression by intervening at the molecular level. Furthermore, targeted therapies can significantly reduce the risk of off-target and adverse effects compared to conventional treatments by acting specifically on the molecular targets implicated in the pathogenesis process (18, 19). This safety profile is particularly advantageous in a patient population vulnerable to side effects or complications due to age or comorbidities.

The RAS-RAF-MEK (mitogen-activated protein kinase kinase)-ERK (extracellular signal-regulated kinase) pathway plays a vital role in maintaining the homeostasis of the heart and blood vessels. Dysregulation of this signaling cascade has been linked to vascular and cardiac pathological remodeling, which contributes to the onset and progression of CVD. Recent and ongoing research has provided insights into potential therapeutic interventions targeting the RAS-RAF-MEK-ERK pathway to ameliorate cardiovascular dysfunction. This approach has been promoted by developing modern small-molecule drugs targeting the MEK kinase with high specificity and selectivity, minimizing unwanted off-target effects.

In this review article, we will first (a) highlight the roles of the MEK-ERK pathway in maintaining cardiovascular system health and (b) describe how dysregulation contributes to the onset or progression of pathological cardiovascular conditions. Then, we will (c) illustrate the therapeutic potential of targeting this pathway using MEK inhibitors (MEKI) in preclinical CVD models and clinical studies. Finally, we will (d) share some considerations about the advantages and challenges of using MEKI in cardiovascular medicine, including possible future developments of this therapeutic strategy.

## 2 RAS-RAF-MEK-ERK pathway

The RAS-RAF-MEK-ERK pathway is a fundamental signaling cascade within the cells that plays a crucial role in various cellular processes, including proliferation, growth, differentiation, motility, and survival. The pathway transmits signals from the cell surface to the nucleus and comprises several key components, each with specific functions (20, 21).

**Figure 1:**
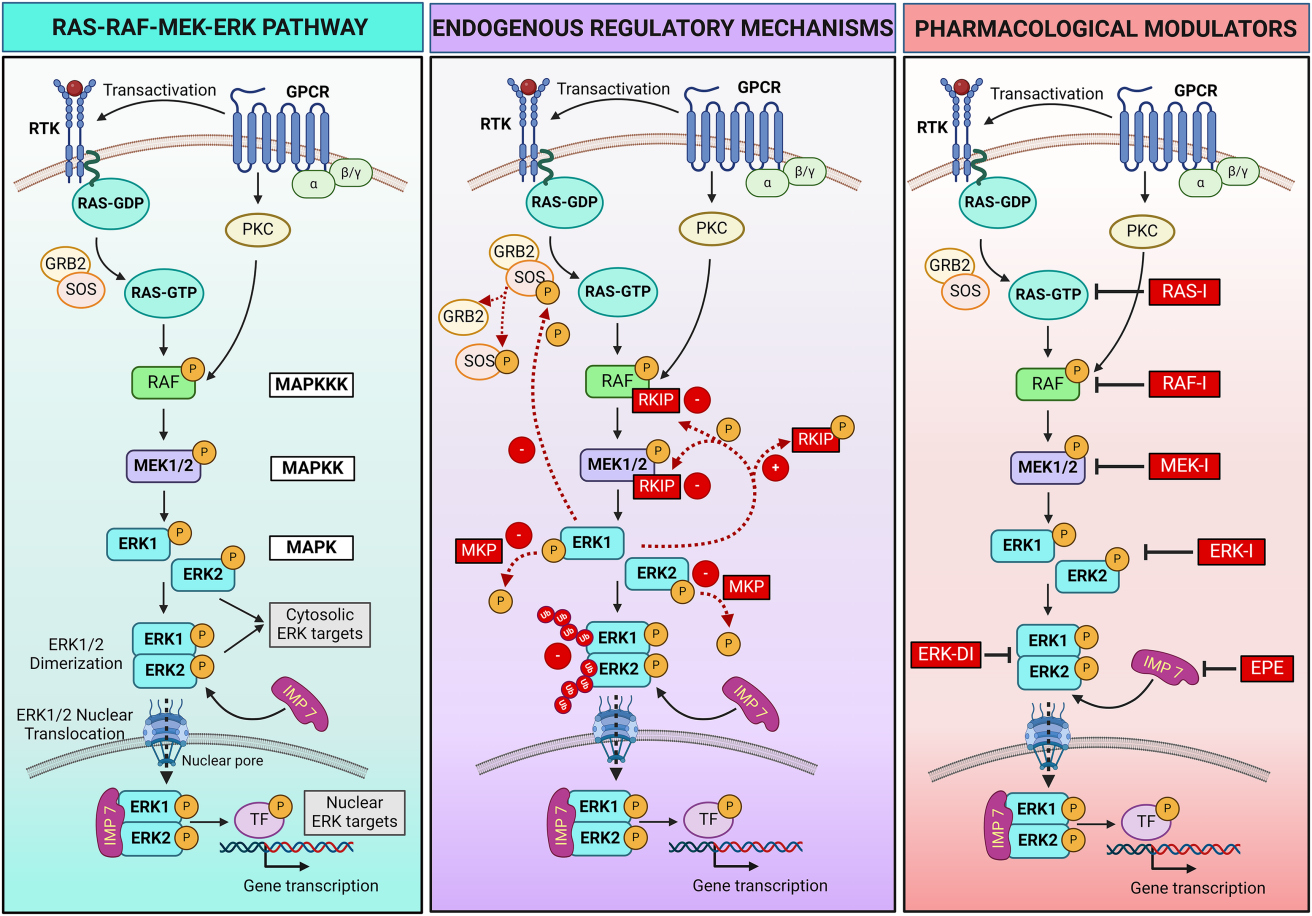
RAS-RAF-MEK-ERK pathway signaling and regulatory mechanisms. Left panel: schematic showing the RAS-RAF-MEK-ERK pathway triggered by activation of receptor tyrosine kinase (RTK) or hormone and chemokine G protein-coupled receptors (GPCRs) following extracellular ligands binding. Central panel: endogenous positive (+) and negative (–) mechanisms that regulate the pathway activation. Ub, ubiquitin. Right panel: Pharmacological strategies can be employed to regulate the pathway. Specific inhibitors (**I**) bind and block each kinase of the pathway (RAS, RAF, MEK, ERK), preventing the phosphorylation/activation of the downstream kinase. An ERK dimerization inhibitory peptide (ERK-DI) can be used to prevent the ERK dimer formation, while a myristoylated phosphomimetic peptide (EPE) can be used to block the interaction between ERK and its protein shuttle Importin 7 (IMP 7), thus blocking the ERK nuclear translocation across the nuclear pores. For more details, refer to Section 2 of the review article. Created with BioRender.com.

At its core, the RAS-RAF-MEK-ERK pathway consists of a three-tiered kinase cascade (Figure 1). The top tier is initiated by a family of cell surface receptors, including growth factor receptor tyrosine kinases (RTKs) and hormone and chemokine G protein-coupled receptors (GPCRs), which respond to extracellular ligands. Upon ligand binding, these receptors adopt different strategies to recruit the first set of intracellular serine/threonine protein kinases, known as RAF kinases (A-, B- and C-RAF, also called MAPKKK - mitogen-activated protein kinase kinase kinase). Growth factor binding induces RTK autophosphorylation, which promotes the recruitment of adaptor molecules like GRB2 to the plasma membrane. GRB2 recruits and forms a complex with the RAS guanine nucleotide exchange factor SOS (Son of Sevenless), resulting in RAS activation and the recruitment of RAF kinases (20). Instead, hormone or chemokine binding triggers the transactivation of RTKs or recruitment of intracellular protein kinase C (PKC), which in turn activates RAF. RAF kinases, when activated, phosphorylate and activate the second tier, the mitogen-activated protein kinase kinase (MAPKK), specifically MEK1 and MEK2. MEKs are dual-specificity serine/threonine and tyrosine protein kinases that, once activated, phosphorylate the third-tier proteins, the extracellular signal-regulated kinases (ERKs, also called MAPK) (22).

ERK1 and ERK2 are the primary members of the ERK family. Upon activation, phosphorylated ERK (P-ERK) phosphorylates hundreds of cytosolic targets, including cytoskeletal elements (e.g., microtubules, actin, myosin light chain kinase) to control cell motility (23–25) and mitochondrial protein Bcl-2 to promote cell survival (26). Moreover, ERK1 and ERK2 can assemble in dimers (ERK1/2). When discovered in 1998, dimers were thought indispensable for ERK translocation into the nucleus (27). This hypothesis was corroborated by two recent studies describing the assembly of ERK1/2 dimers as an essential event for nuclear translocation and ERK interaction with its nuclear substrates. Specifically, the mechanism involves the autophosphorylation of ERK1/2 on Threonine 188 occurring exclusively within the ERK dimers and that is required to initiate nuclear translocation (28, 29). In the nucleus, ERK dimers phosphorylate transcription factors, leading to changes in gene expression. Examples of transcription factors regulated by ERK1/2 include Signal transducer and activator of transcription 3 (STAT3, which controls the cell cycle by regulating the transcription of the *CCND1* gene encoding cyclin D) (30), ETS Like-1 protein (ELK1, which controls cell differentiation) (31), and the oncogene c-MYC (which promotes cell growth and survival) (32). The role of ERK dimers, however, remains uncertain. According to recent contrasting findings, ERK dimers control cytosolic but not nuclear substrates and are not required for nuclear translocation, suggesting nuclear functions are due to ERK monomers (33–35).

### 2.1 Regulation of the RAS-RAF-MEK-ERK pathway

Multiple endogenous mechanisms fine-tune the RAS-RAF-MEK-ERK pathway to ensure that signaling is turned on and off in a timely manner to control cellular processes (Figure 1). Negative and positive feedback loops, scaffold proteins, and a balance between activating kinases and inactivating phosphatases play a critical role in modulating the intensity and duration of signaling. The ERK pathway can also be regulated through crosstalk with other signaling pathways. In addition, pharmacological strategies can be adopted to regulate the RAS-RAF-MEK-ERK pathway when needed, for example, in pathological settings.

#### 2.1.1 Endogenous negative regulatory mechanisms

The RAF kinase inhibitory protein (RKIP) is one negative regulatory protein. RKIP binds to RAF and MEK, preventing their association and, therefore, MEK phosphorylation (36, 37). A negative feedback mechanism occurs by phosphorylation of SOS by cytoplasmic P-ERK, resulting in the dissociation of SOS from GRB2 and failure to activate RAS (38, 39). Additionally, several phosphatases, such as MAPK phosphatases (MKPs), protein phosphatase 2A (PP2A), and dual-specificity phosphatases (DUSPs), work to deactivate the pathway by dephosphorylating key components (40, 41). Moreover, the duration of the activation of critical components of the pathway is tightly controlled by the ubiquitin-mediated degradation of the activated kinases, ensuring that signaling is turned off when no longer needed (42, 43).

#### 2.1.2 Endogenous positive regulatory mechanisms

Ligands binding RTKs and GPCRs positively regulate the pathway by inducing its activation. These ligands include multiple growth factors (e.g., epithelial cell-derived growth factor and hepatocyte-derived growth factor), hormones, chemokines, and neurotransmitters. A positive feedback mechanism results from the phosphorylation of RKIP by P-ERK, leading to dissociation of RKIP from RAF and MEK and continuous MEK activation (44).

#### 2.1.3 Scaffold proteins and subcellular regulation

The ERK pathway is not only regulated by the phosphorylation status of the kinases composing the pathway, but also by the spatial compartmentalization of the ERK signal within the cell. Indeed, ERK activation at different subcellular locations leads to specific cellular responses. Multiple ERK scaffolding proteins bind ERK and determine the spatial localization and selectivity of its signal (27, 33, 35, 45, 46). Some scaffold proteins act as anchors to retain ERK within the cytosol, facilitating ERK physical interaction with cytosolic substrates and their phosphorylation to regulate cellular functions such as survival. Examples of these scaffolds include KSR1 (kinase suppressor of RAS), β-arrestin, and SEF. Specific scaffold proteins regulate ERK activation in selective cellular compartments, for example, KSR on the plasma membrane, MEK partner 1 (MP-1) at endosomes, SEF at the Golgi apparatus, and Paxillin at focal adhesions (47). Another protein (Importin 7) behaves like a shuttle that transports ERK across the nuclear pores, allowing its translocation into the nucleus and interaction with nuclear targets to control gene expression (48, 49). Scaffold proteins also serve as a *dimerization platform* to facilitate ERK monomer interaction and dimer assembly (35).

#### 2.1.4 Pharmacological modulators

Two synthetic peptides were recently developed and tested in preclinical studies—mostly *in vitro*—to modulate the final part of the pathway. The ERK dimerization inhibitory peptide (ERK-DI, DEL-22379) prevents ERK dimer assembly and, thereby, its nuclear translocation (29, 50). Instead, a myristoylated phosphomimetic peptide (EPE peptide) specifically blocks the ERK dimer interaction with Importin 7 and, thereby, its nuclear translocation (51). An advantage of both these approaches is that they do not inhibit ERK phosphorylation, thus conserving the cytosolic ERK activity and safeguarding cell viability and survival while avoiding uncontrolled cell growth or proliferation due to nuclear P-ERK1/2 dimers activity (29, 50–53).

Pharmacological inhibitors that target and turn off key intracellular components of the pathway include RAS, RAF, MEK, and ERK inhibitors, all under clinical development. These compounds bind the different kinases of the pathway and inhibit their enzymatic activity, thus preventing phosphorylation/activation of the downstream target kinase (54, 55). This review will focus on pharmacological inhibition of MEK1/2 activity using MEKI.

## 3 ERK signaling in cardiovascular health

The ERK signaling plays a vital role in regulating various physiological functions of the adult cardiovascular system, helping maintain myocardial and blood vessel homeostasis.

### 3.1 Myocardial homeostasis

In cardiomyocytes, ERK signaling mediates the responses to external stimuli and ensures cell survival (56). Several studies showed that ERK activation is an essential adaptive mechanism that promotes cardiomyocyte survival during early stress (57–60). Moreover, ERK signaling controls the cellular response to mechanical forces (61), critical for an organ that contracts cyclically throughout life.

ERK signaling also participates in controlling the heart's electrical conduction system (62). By influencing the behavior of specialized cardiac cells that regulate heart rhythm, a balanced ERK activation helps monitor electrical signals, contributing to normal cardiac conduction and rhythm (62).

### 3.2 Angiogenesis and vascular homeostasis

The ERK signaling is essential for angiogenesis sprouting by mediating endothelial cell (EC) proliferation, migration, and tube formation. *In vitro*, pretreatment of monocultures of ECs with compounds restraining the ERK activation resulted in decreased EC network formation in angiogenesis assays (63–65). ERK ensures vascular stabilization (66). ERK signaling maintains the integrity of the EC barrier by regulating the expression of proteins involved in cell-cell adherence junctions (67), thus preventing the extravasation of blood components. ERK signaling also modulates the response of ECs to blood flow-induced mechanical forces, including shear stress (68). This function of intimal ECs is essential to adapt to changes in blood flow patterns and to maintain vascular wall homeostasis. Moreover, ERK activity regulates the expression and production of antioxidant enzymes in ECs in response to oxidative stress (69, 70). Activated ERK regulates the nitric oxide synthase (NOS) enzyme responsible for endothelial NO production (71–73). NO is a potent vasodilator and vascular tone modulator.

The vascular wall comprises different cell types, mainly ECs and pericytes, and vascular smooth muscle cells (VSMCs) in arteries and veins. The ERK signaling is central in determining these cells’ phenotypes and responses to extracellular stimuli. For example, the ERK signaling preserves the undifferentiated antigenic phenotype of cardiac pericytes (64). Pericytes wrap around blood vessels in direct contact with ECs and are fundamental to safeguarding vascular integrity and function (74). Balanced ERK activity is required to prevent dedifferentiation of contractile VSMCs towards a proliferative synthetic phenotype, a hallmark of vascular pathologies (64, 75, 76). ERK regulates vascular tone by influencing the contraction and relaxation of VSMCs within the tunica media, thus inducing vasoconstriction or vasodilation as required to control blood pressure and the delivery of proper amounts of oxygen and nutrients to tissues (77).

## 4 ERK signaling in cardiovascular disease

The ERK signaling is essential to turn on compensatory mechanisms to control the cellular response to stress during the early phases of diseases. Moreover, ERK activates key reparative mechanisms after tissue injury. Nonetheless, research has suggested that aberrant activation or perpetuation of this signaling can trigger cellular and molecular mechanisms underlying CVD. Here, we summarize the role of ERK signaling in the context of three primary cardiovascular conditions.

### 4.1 Coronary artery disease

Coronary artery disease (CAD) represents a complex heart condition characterized by a narrowing of the coronary arteries that supply blood to the heart muscle. MI, commonly referred to as a heart attack, is a life-threatening, acute event that occurs when a blood clot blocks the blood flow in one of the coronary arteries, causing the death of the myocardial region perfused by that artery. Atherosclerosis, the progressive buildup of lipid plaques within arterial walls, represents a significant risk factor for developing CAD. The ERK pathway plays a central role in the pathogenesis and progression of CAD, influencing all the phases of the disease, from atherosclerosis and the initial inflammatory response after MI to later tissue repair and remodeling.

#### 4.1.1 Spatiotemporal pathway activation

In rodent reperfused MI models, cardiac P-ERK levels are raised after ischemia and a few hours post-reperfusion (78–80). A study from Yeh et al. using a murine MI model with permanent left anterior descending coronary artery (LAD) occlusion demonstrated that ERK1/2 phosphorylation is triggered early after MI in the infarct, where it remains elevated up to 12 weeks post-MI. In contrast, ERK activation is delayed in the remote myocardium and reaches its peak after 12 weeks (81). This attractive spatiotemporal activation gradient could be a physiological mechanism adopted by the heart to activate reparative mechanisms where mostly needed after MI, for example, to maximize the recruitment of immune cells to the ischemic area.

#### 4.1.2 Cardioprotective effects of ERK signaling and aging

After MI, ERK signaling is required to protect the myocardium from ischemia and reperfusion injury. Benefits include protection from apoptosis and preservation of volume and pressure hemodynamic function (57). With aging, the heart's ERK activity physiologically declines. While P-ERK levels rise in the hearts of young mice quickly after MI as part of a physiological protective mechanism, this response is lost in aged mice (82). Interestingly, MEK overexpression in the hearts of aging mice via viral transduction of a constitutively active MEK protein activated ERK and protected the hearts against MI injury. Benefits included increased left ventricular ejection function, improved mitochondrial function, decreased myocardial apoptosis, and lower serum concentrations of markers of myocardial injury (lactate dehydrogenase and creatine kinase) (82).

#### 4.1.3 Cell viability

During the acute phase of MI, when blood flow to a heart region is blocked, ERK signaling can promote cardiomyocyte survival by triggering pro-survival pathways and help limit myocardial damage. Each single cardiomyocyte is surrounded by several capillaries in the heart, ensuring the delivery of oxygen and nutrients. By upregulating vascular endothelial growth factor A (VEGFA) expression and boosting EC migration and angiogenesis, ERK signaling contributes to preserving cardiomyocyte survival in the ischemic area at risk of death (83). However, aberrant ERK signaling results in abnormal cardiomyocyte growth, a central mechanism in the development of pathological cardiac hypertrophy (see Section 4.2).

ERK signaling post-MI also controls the viability of non-cardiomyocyte cells, including fibroblasts, myofibroblasts, macrophages, and other immune cells, essential for forming granulation tissue in the acute stages post-MI. Granulation tissue is a prerequisite for harmonized heart repair following MI by promoting the formation of a thick, stable scar (84).

#### 4.1.4 Angiogenesis

Angiogenesis is vital for restoring blood flow to the infarcted area by promoting the formation of new microvessels, with ERK signaling being a core part of this process. VEGFA is a master proangiogenic factor indispensable for blood vessel formation and whose expression is physiologically increased under hypoxia (85). One mechanism by which ERK signaling activates EC migration and promotes angiogenesis involves the upregulation of the *VEGFA* gene transcription by enhancing the action of the hypoxia-inducible factor-1 (HIF-1) (83). ERK pathway activation also promotes the maturation of collateral blood vessels (a phenomenon called arteriogenesis) (86), aiding in the quick restoration of blood supply to the damaged heart tissue. However, while these processes may initially be adaptive to increase blood supply to the heart, uncontrolled angiogenesis can develop disorganized and leaky vessels, further impairing cardiac function.

Nonetheless, there are controversial reports regarding the role of ERK signaling in post-MI vascularization. On the one hand, ERK is reportedly required to promote angiogenesis, blunted by pharmacological inhibition of ERK activity (63). On the other hand, ERK phosphorylation prevention promoted the ischemic heart’s vascularization by increasing cardiac pericyte proangiogenic activity and differentiation into VSMC-like cells (64). These differences might be due to the different therapeutic regimens and influenced by the spatiotemporal dynamics of ERK activation occurring after MI.

In atherosclerosis, activated ERK signaling stimulates the secretion of the proangiogenic factor VEGFA, promoting angiogenic activity in the atheromatous lesion (87–89). Active angiogenesis within atherosclerotic plaques is harmful, contributing to intraplaque hemorrhage, plaque expansion, destabilization, and rupture (90, 91).

#### 4.1.5 VSMC phenotype

ERK signaling promotes the proliferation and dedifferentiation of contractile VSMCs into the synthetic phenotype during the early and advanced stages of atherosclerotic plaque formation (92, 93). Activated ERK stimulates the expression of biglycan in immature VSMCs (94). Biglycan is a small leucine-rich repeat ECM proteoglycan that favors VSMC proliferation and migration (95). Therefore, ERK induces the migration and proliferation of VSMCs, promoting the formation of a fibrous cap over the plaque (96). This cap can destabilize the plaque, increasing the risk of rupture and thrombosis. The ERK pathway was also involved in the osteoblast differentiation of VSMCs *in vitro*, which can lead to vascular calcification and deterioration of CAD (97).

#### 4.1.6 Inflammation and endothelial dysfunction

ERK signaling favors the inflammatory response during MI. ERK activation in immune cells stimulates the release of proinflammatory cytokines and chemokines, like tumor necrosis factor (TNF), interleukin (IL)-1β, and IL-6 (98), contributing to inflammation in the infarcted area. While inflammation is a natural part of the healing process and a prerequisite for cardiac repair, excessive or persisting inflammation can worsen tissue injury.

During percutaneous transluminal balloon angioplasty, ERK signaling is rapidly activated in intimal ECs due to the physical injury caused by the balloon and in VSMCs due to their response to the inflammatory reaction triggered by the stent fitting. Activated VSMCs become more proliferative and migratory and produce higher amounts of ECM, posing the basis for neointima formation (99–102). This latter is a harmful process that leads to coronary artery restenosis and repeated ischemic events.

Inflammation and endothelial dysfunction are early events in the atherogenesis process. Activated ERK induces expression of cyclooxygenase-2 and secretion of proinflammatory prostaglandin E (103). ERK signaling is also involved in the formation of neutrophil extracellular traps (NETs). NETs amplify the recruitment of immune cells and stimulate the formation of atheromatous plaques (104, 105). Activated ERK signaling is strongly associated with endothelial-to-mesenchymal transition (EndMT). EndMT is involved in the pathological process of atherosclerosis by inducing inflammatory response, intimal hyperplasia, and secretion of ECM proteins (106). During inflammation, ERK signaling activation is required in ECs to attract and facilitates the trans-endothelial migration of immune cells (leukocytes) mediated by the upregulation of adhesion molecules (107). This inflammatory response contributes to the initiation of atherosclerotic plaques.

Macrophages play a central role in atherosclerosis by accumulating lipids and undergoing a transformation into foam cells, a hallmark of atherosclerotic lesions (108, 109). ERK signaling is involved in macrophage activation, which leads to the uptake of oxidized low-density lipoprotein cholesterol and foam cell formation (110, 111). Foam cells contribute to the atheromatous plaque progression and complications (112). ERK signaling also contributes to the recruitment of macrophages and phagocytic cells to the site of the atheromatous plaque and the perpetuation of the inflammatory process.

#### 4.1.7 ECM remodeling, fibrosis, and scar formation

As the heart heals in the post-MI phase, ERK signaling plays a role in tissue repair and fibrosis. This pathway stimulates the proliferation of fibroblasts, which deposit collagen and other ECM components, contributing to fibrosis (113). While scar formation is crucial for maintaining structural integrity and avoiding heart rupture, excessive myocardial fibrosis can lead to pathological remodeling, ultimately impairing cardiac function and promoting the transition to heart failure.

The ERK pathway also influences the ECM remodeling within the arterial wall. Overactivated ERK stimulates the expression and production of matrix metalloproteinases 2 and 9 (MMPs) (114). MMPs have a crucial role in the progression of atherosclerosis (96). MMPs contribute to the degradation of ECM, facilitating VSMC migration and proinflammatory cell infiltration into the atheromatous plaque (115). Overexpression of MMPs can weaken the fibrous cap, making it more susceptible to rupture (96, 115).

### 4.2 Heart failure and cardiac hypertrophy

Heart failure is a condition in which the heart loses its ability to pump blood to the whole body efficiently. Heart failure can be the result of adverse post-MI heart remodeling or the consequence of pressure or volume overload-induced cardiac hypertrophy, perhaps due to hypertension or valvular heart disease. Cardiac hypertrophy is characterized by the enlargement of cardiomyocytes, which results in the enlargement of the whole organ. While limited hypertrophy is a normal response to increased workload (for example, physiological hypertrophy observed in athletes in response to high-intensity exercise training), pathological hypertrophy leads to heart dysfunction.

ERK signaling plays a significant role in the development and progression of heart failure. ERK activation was demonstrated in cardiac tissue of both human patients with heart failure (116) and preclinical animal models of cardiac hypertrophy and heart failure induced by transverse aortic constriction (TAC) surgery (117, 118). In preclinical models, ERK activation was observed at different times post-TAC surgery, spanning from a few minutes up to several days (117, 118), thus suggesting ERK signaling might play distinct roles in heart failure according to the dynamics of temporal activation.

#### 4.2.1 Cardiomyocyte growth and viability

ERK signaling is involved in the initiation and progression of cardiac hypertrophy (14). In the initial phases of pressure or volume overload, ERK activates a compensatory hypertrophic response by enhancing myocardial contraction to help the heart adapt to the increased mechanical strain and preserve cardiac function (119). ERK promotes the growth of cardiomyocytes, increasing cell size through mechanisms like protein synthesis and sarcomere organization (120, 121). Pressure overload stimulates cardiomyocyte surface receptors, including stretch-sensitive membrane integrins (122) and GPCRs (123). Stimulation of these receptors triggers the ERK pathway activation mediated by intracellular scaffold β-arrestin (124). Activated ERK dimers translocate into the nucleus and influence the expression of genes involved in cell growth and survival (125). However, while this early adaptive response aims to compensate for cardiac stress, prolonged activation of ERK signaling leads to a transition to pathological or maladaptive cardiac hypertrophy and adverse heart remodeling, ultimately promoting heart failure. Dysregulated ERK signaling also impairs cardiomyocyte viability, leading to a loss of functional heart muscle cells and contributing to contractile dysfunction.

#### 4.2.2 ECM remodeling and fibrosis

ERK signaling is involved in ECM remodeling and fibrosis, hallmarks of cardiac hypertrophy and heart failure. Activation of ERK signaling by external stimuli such as Endothelin-1 (released by ECs exposed to high pressure) is required for the proliferation of cardiac fibroblasts and their differentiation into myofibroblasts, which deposit excessive amounts of collagen and other ECM components in the heart, contributing to myocardial fibrosis (113, 126). ERK signaling is also involved in controlling the release of MMP2 and MMP9 and tissue inhibitors of metalloproteinases (TIMPs), maintaining the balance between matrix synthesis and degradation (127, 128). When this process becomes unbalanced, it leads to excessive deposition of collagen and other ECM components in the heart. The fibrotic tissue surrounding cardiomyocytes can interfere with the conduction of electrical signals in cardiomyocytes, leading to an increased risk of arrhythmias.

#### 4.2.3 Inflammation

As described in the context of CAD, ERK signaling leads to the recruitment of immune cells and the release of proinflammatory cytokines and chemokines (98), contributing to heart inflammation. Perpetual inflammation promotes fibrosis and damages the cardiac tissue, thus worsening heart failure.

#### 4.2.4 Hypertrophic cardiomyopathy (HCM)

Dysregulation of ERK signaling is linked to HCM (129), a genetic condition characterized by abnormal thickening of the heart muscle, which can cause arrhythmias, heart failure, and sudden cardiac death. In HCM patients, mutations of genes associated with the ERK pathway led to unbalanced ERK activation and cardiomyocyte growth. In *in vitro* experiments with patients induced pluripotent stem cells (iPSC)-derived cardiomyocytes, ERK activity was required to develop the pathological phenotype, while its inhibition rescued the HCM phenotype (129).

### 4.3 Aortic aneurysm

An aortic aneurysm is an irreversible and progressive dilation of a portion of the aorta to greater than 50% of the standard diameter. The aorta walls undergo structural remodeling and become progressively thinner and weaker. If untreated, the aneurysm can rupture with fatal consequences for the patient. Recent research suggested that ERK signaling plays a role in the pathogenesis and progression of aortic aneurysms. Accordingly, experimental findings showed that ERK phosphorylation is increased in the aorta of both humans and rodents with aortic aneurysms (130, 131). In a mouse model of Marfan syndrome, ERK1/2 signaling was the driver of aortic aneurysm formation (132).

#### 4.3.1 Adventitia remodeling

Vasa vasorum are small arterioles that supply oxygen and nutrients to the adventitial layer and to the external part of the medial layer of the aorta. Billaud et al. showed that aortic aneurysm is associated with inhibited angiogenic activity in the aortic adventitia. An altered balance of pro- and anti-angiogenic factors was reflected in low vasa vasorum density (133). The MEK/ERK is the principal pathway involved in regulating the angiogenic activity of ECs and pericytes, essential cellular components of vasa vasorum. Dysregulated ERK signaling was associated with an impaired angiogenic function, which ultimately affects the perfusion and weakens the structure of the outer layers of the aorta, contributing to aneurysm progression (64, 83, 134).

#### 4.3.2 VSMC phenotypic switch

VSMCs are the main component of the medial layer of the aortic wall (135). Impairment of VSMC function is associated with the disruption of the wall structural integrity that, together with the high intra-luminal pressure, leads to aneurysm formation (136). Several studies illustrated the contribution of ERK signaling to VSMC dysfunction. Overactivity of the ERK pathway stimulates VSMC phenotypic change, favoring the transition from a contractile to a synthetic phenotype, which harms the aortic wall integrity and reduces its elasticity (137–139). Synthetic VSMCs are more proliferative and secret more ECM proteins, contributing to aortic wall remodeling, enlargement, and dysfunction (140, 141). Oxidative stress is a primary contributing factor in the pathogenesis of aortic aneurysm. By inducing ERK signaling activation, oxidative stress favors the VSMC phenotypic switch (142, 143).

#### 4.3.3 ECM remodeling

ECM is a critical structural component of the medial and adventitial layers of the aorta. Activation of MMP2 and MMP9 and the arising disruption or loss of ECM significantly affect the integrity of the aortic wall and contribute to aneurysm formation (144–146). In VSMCs, ERK stimulates the secretion and activity of MMP-2 and MMP-9, which degrade and break down essential components of the ECM, including collagen and elastin (131, 147, 148). ERK silencing in aortic VSMCs resulted in significantly reduced production of MMP2 and MMP9 (131). Moreover, activated ERK stimulates the production of disorganized and dysfunctional fibronectin and collagen fibers by synthetic secretory VSMCs (138). Impaired ERK signaling causes abnormal patterns of cross-linking of collagen fibers, which play a role in the formation of aneurysms in the context of congenital diseases encompassing, among others, aortic manifestations (149, 150). Excessive fibronectin deposition promotes fibrosis, which leads to stiffening and impaired mechanical properties of the aortic wall (151).

#### 4.3.4 Inflammation

Chronic inflammation and MMP2 and MMP9 activation are the hallmark pathological process of aortic aneurysms (145, 152, 153). ERK activation mediates the inflammatory response by recruiting neutrophils, inducing the production of proinflammatory cytokines and chemokines, and activating macrophages (154–156). Aortic VSMCs exposed to IL-1β, or TNF showed increased MMP2 or MMP9 activation mediated by a mechanism involving amplified ERK phosphorylation (148, 157), thus promoting aneurysm progression.

## 5 MEK inhibitors

As described above, several cardiovascular conditions are characterized by aberrant activation of ERK signaling. These findings inspire the development of novel therapeutic approaches to correct ERK signaling at physiological levels. One effective strategy is to prevent ERK phosphorylation/activation by blocking its upstream activator—MEK1/2—using MEKI.

MEKI are modern small-molecule drugs that bind specifically to MEK and induce conformational changes that lock the kinase in a catalytically inactive state (158). As a result, the downstream ERK is not phosphorylated and remains inactive. Because ERK signaling is aberrantly activated in at least one-third of malignant tumors, MEKI are gaining considerable attention and momentum in cancer therapy for their ability to stop proliferation and induce the death of cancer cells.

MEKI are relatively modern drugs, with the first compound being described in 1995 (159). Over the years, several small molecules have been synthesized with increasing selectivity for MEK. Compared with the first generation of MEKI, the new-generation MEKI are highly selective and bind to a site near the adenosine triphosphate (ATP)-binding pocket uniquely present on MEK proteins, thus avoiding off-target effects on other kinases. Therefore, MEKI act through a non-ATP competitive allosteric mechanism (158, 160). This last characteristic is an advantage to maximize the drug action without competing with high concentrations of ATP present within the cells. Most MEKI are still in the preclinical development phase, like U0126 and PD98059. Only in 2013 was Trametinib first approved for the treatment of cancer patients. In recent years, additional MEKI were approved by the Food and Drug Administration (FDA) or the European Medicine Agency (EMA) for clinical studies in cancer patients. Approved MEKI include Cobimetinib, Selumetinib, Binimetinib, Encorafenib, Refametinib (RDEA-119), Pimasertib, and Mirdametinib (PD0325901).

MEKI are ideal for targeted therapy to avoid off-target effects due to the drug binding to other cellular kinases. This characteristic makes MEKI safer than multitarget drugs that act on multiple cellular proteins more broadly, like tyrosine kinase inhibitors (TKI). Two examples of TKI are Sunitinib and Imatinib, which ultimately cause cardiovascular toxicity and heart failure (161–164). So far, MEKI have also shown better tolerance and success than ERK1/2 inhibitors (ERKI) in clinical trials on cancer patients. In these patients, ERKI showed scarce efficacy and quick development of drug resistance due to the compensatory activation of other functionally redundant members of the large MAPK family (like ERK5) after the inhibition of ERK1/2 (55). Another reason for the short-duration efficacy of ERKI is their mechanism of action. Since most ERKI are ATP-competitive and must compete with ATP for binding to ERK, the concentration of ERKI within the cells should ideally be superior to that of ATP for optimal effect. Last, because the ATP-binding pocket is highly conserved in several kinases, ERKI do not selectively bind to ERK1/2 but also to other kinases, provoking undesired off-target effects (55).

While MEKI are primarily explored in the context of cancer therapy, there is a growing interest in testing these drugs to treat or prevent the progression of cardiovascular conditions characterized by dysregulated ERK activation.

The advantages of repurposing cancer drugs for the treatment of CVD are multiple. Among them, we find (a) a lower risk of failure for safety, (b) limited development costs compared with new drugs, (c) extensive knowledge of side effects, and (d) quicker clinical application (165–167). Not less important, because the development of new cardiovascular drugs is slow (e.g., only two new drugs were approved by the FDA for cardiovascular applications in 2020 compared with 36 drugs for cancer), repurposing existing drugs would allow faster identification of new treatments for cardiovascular patients (165).

## 6 Therapeutic potential of MEKI for cardiovascular disease

Exploiting MEKI as a potential therapeutic in cardiovascular medicine remains a novel and largely unexplored field. This is likely due to these drugs being still in the clinical development phase for the treatment of some cancers and more extensive information about their safety and long-term side effects in cancer patients is needed. Importantly, additional studies will be required to establish whether MEKI application in cardiovascular patients would be safe, both for short- and long-term treatments. Here, we discuss possible applications of MEKI for the management of CVD, based on current knowledge collected during preclinical and clinical studies.

### 6.1 Preclinical cardiovascular research with MEKI

**Figure 2:**
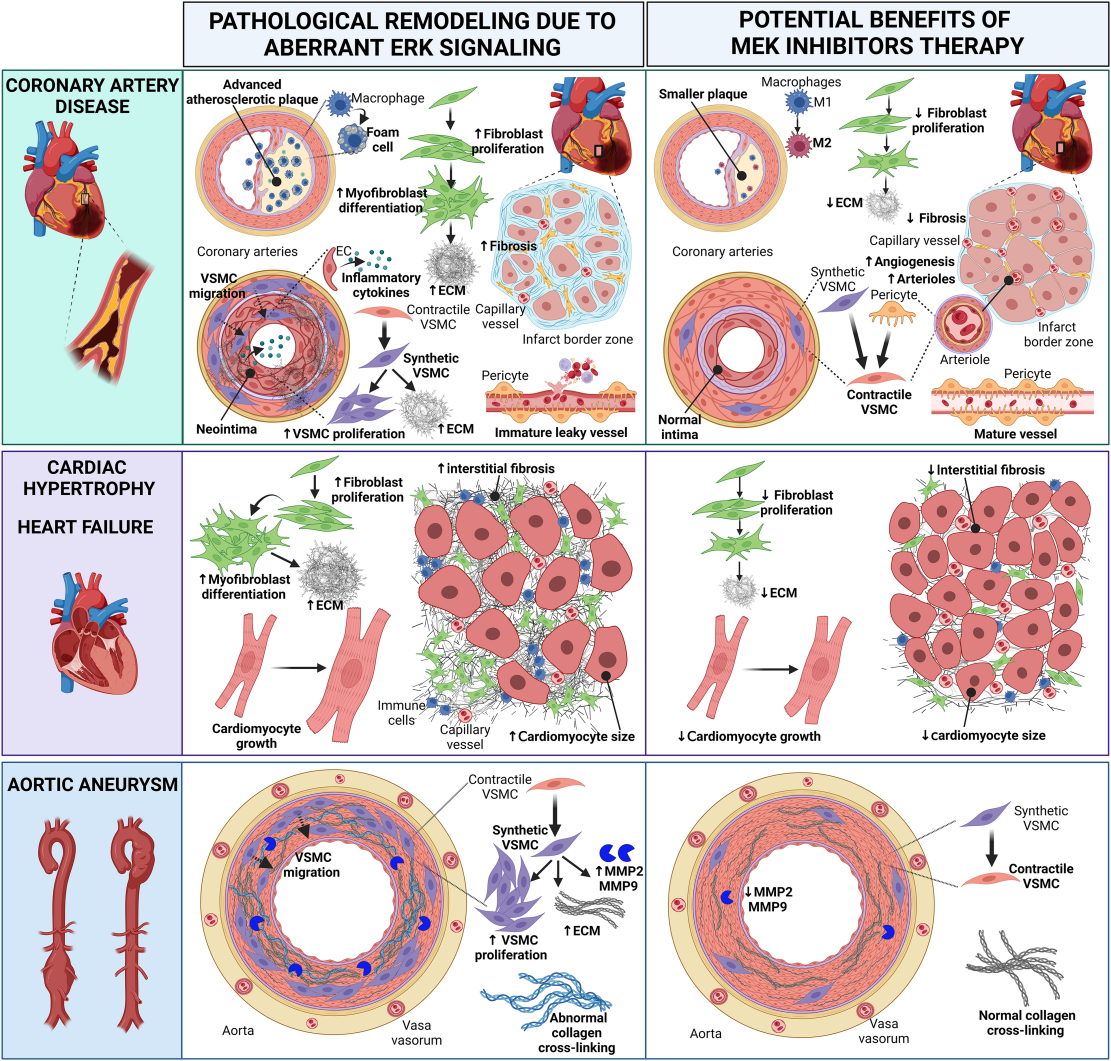
Cartoon summarizing the pathological heart and vascular remodeling caused by dysregulated ERK activity and potential therapeutic benefits of MEK inhibitors (MEKI). In *coronary artery disease*, MEKI slow the progression of atherosclerotic plaques and reduce foam cell formation; favor the switch of M1 macrophages into the anti-inflammatory M2 phenotype; decrease fibroblast proliferation, thus preventing excessive ECM synthesis; promote the differentiation of synthetic migratory VSMCs into contractile VSMCs, contrasting neointima formation and vascular fibrosis; induce the differentiation of pericytes into contractile VSMC-like cells, aiding the maturation of new arterioles; promote the revascularization of the infarct border zone, enhancing capillary and arteriole density and reducing interstitial myocardial fibrosis; promote the maturation of functional neovessels. In *cardiac hypertrophy* and *heart failure*, MEKI decrease fibroblast proliferation and ECM synthesis, thus reducing interstitial myocardial fibrosis; attenuate cardiomyocyte growth. In *aortic aneurysms*, MEKI promote the differentiation of synthetic VSMCs into contractile VSMCs, thus reducing the synthesis of ECM proteins and the release of MMP enzymes; prevent abnormal collagen crosslinking. Both these events help preserve the aortic wall integrity. EC, endothelial cell; ECM, extracellular matrix; MMP, matrix metalloproteinase; VSMC, vascular smooth muscle cell. Created with BioRender.com.

Preclinical studies with *in vitro* cell cultures and animal models of CVD have laid the foundation for further exploring the potential use of MEKI to prevent or attenuate pathological heart and vascular remodeling (Figure 2). As follows, we summarize the *in vivo* studies with MEKI. Additional details for these studies are provided in Table 1.

**Table 1:** Summary of preclinical studies with MEKI in CVD *in vivo* models.

Pathological model	Species	MEKI tested	Dosage	Starting time of treatment	Treatment duration and frequency. Administration route	Effects on cardiovascular physiology and pathology (compared with vehicle)	Effects at the cellular level	Reference
Chronic MI (also tested on healthy heart)	Mouse	PD0325901	10 mg/kg/day	3 days post-MI	14 days once daily (Oral)	Improved LV vascularisation, perfusion, and function. Smaller infarct scars	In *healthy hearts*, induced PC differentiation into contractile cells. No changes in CM and vascular cells apoptosis, fibroblast density and differentiation	Avolio et al. (64)
Reperfused MI	Rat	PD0325901 (+oleuropein)	3 mg/kg/day	Preventive treatment 2 days pre-MI	2 days (Oral)	Smaller infarct scars. Reduced serum creatine kinase MB and lactate dehydrogenase levels	Inhibited caspase-3 activity. Anti-inflammatory and anti-oxidative effects	Jin et al. (78)
Ischemic preconditioning + MI	Swine	PD98059 U0126	Local 6.25–50 µM Systemic 5–7.5 mg	Before IPC and post-MI during reperfusion	15–30 min (IV/IM)	Larger infarct scars	Increased CM apoptosis and inhibited CT-1 pro-survival effects	Strohm et al. (79)
Ischemic preconditioning + MI (***ex vivo*** excised hearts)	Rat	PD98059	10 μM	Immediately after ischemia (reperfusion phase)	15 min (during reperfusion)	Increased infarct size	Inhibited IPC protective effect	Hausenloy et al. (80)
Atherosclerosis	Mouse	U0126	3 mg/kg mixed with HFD	With the start of HFD	16 weeks Daily (Oral)	Blunted atherogenic process. Preserved aorta wall's integrity. Reduced lipids accumulation	Reduced macrophage and foam cell formation	Chen et al. (168)
Balloon-induced carotid injury	Rat	PD0185625	200 mg/kg/day	2 days pre-carotid injury	16 days Once daily Oral)	Prevented neointima formation	Inhibited intimal and medial cell proliferation. No change in apoptosis	Gennaro et al. (169)
NF1 Ligation-induced carotid injury	Mouse	PD0325901	5–10 mg/kg/day	7 days pre- or 7 days post-carotid injury	35–21 days Once daily (Oral)	Prevented carotid neointima formation when started before injury. Reduced neointima formation when started after injury	Reduced intimal macrophage infiltration	Stansfield et al. (170)
Cardiac hypertrophy (Activated c-MET expression)	Mouse	Pimasertib	4 mg/kg/day	Post-natal days 21–23	3 days Once daily (IP)	Attenuated hypertrophy Prevented heart failure	Reduced hypertrophy-related gene expression. No changes in apoptosis or collagen formation	Sala et al. (171)
Cardiac hypertrophy (Pressure overload and AAC)	Rat	Selumetinib	1 mg/kg/day	1-week post-AAC	4 weeks Once daily (IP)	Prevented cardiac hypertrophy and fibrosis	Preserved CM size and attenuated cardiac hypertrophy markers	Li et al. (172)
Cardiac hypertrophy (Induced by L-NAME)	Rat	PD98059	5 mg/kg/day	At the same time with L-NAME	8 weeks Once daily (Oral)	Increased systolic BP. Attenuated LVW increase induced by L-NAME	Attenuated CM size increase induced by L-NAME	Sanada et al. (173)
Hypertrophic cardiomyopathy (Noonan Syndrome)	Mouse	PD0325901	5 mg/kg/day	At 4-week age	6 weeks Once daily (IP)	Ameliorated cardiac function. Corrected anatomical defects	Normalized CM size	Wu et al. (174)
Aortic aneurysm (Marfan Syndrome)	Mouse	RDEA119	25 mg/kg/day	At 2-month age	2 months Twice daily (Oral)	Prevented aortic root aneurysm formation. Normalised aortic growth	Rescued medial CT loss	Holm et al. (132)

AAC, ascending aortic constriction; BP, blood pressure; CM, cardiomyocyte; c-MET, hepatocyte growth factor receptor; CT, connective tissue; CT-1, cardiotrophin-1; HFD, high fat diet; IM, intramyocardial; IP, intraperitoneal; IPC, ischemic preconditioning; IV, intravenous; L-NAME, N(G)-nitro-L-arginine methyl ester; LV, left ventricle; LVW, left ventricular weight; MI, myocardial infarction; NF1, neurofibromatosis type 1; PC, pericyte.

#### 6.1.1 Myocardial infarction

Short-term treatment with PD0325901 demonstrated therapeutic efficacy in a mouse MI model with permanent LAD occlusion (64). When started 3 days after acute MI, 2-week daily PD0325901 oral treatment enhanced the capillarization and arterialization of the infarcted area by promoting the differentiation of resident cardiac pericytes into contractile VSMC-like cells and enhancing their angiogenic function. This vascular remodeling improved myocardial perfusion, restrained the expansion of the infarct scar, and preserved cardiac systolic function, improving mice survival (64).

In another study using a rat model of reperfused MI (30 min LAD occlusion + 3 h reperfusion), preventive oral treatment with PD0325901 for 3 days reduced the infarct size and lowered the serum levels of biomarkers of myocardial injury (i.e., creatine kinase MB and lactate dehydrogenase) when assessed at the end of the reperfusion time (78). In this study, the MEKI enhanced the MEK/ERK pathway inhibition and pronounced the cardioprotective effects of oleuropein (a glycosylated seco-iridoid found in green olives) (78).

Two *in vivo* studies demonstrated that ERK signaling activation at reperfusion is indispensable for the protective effects of ischemic preconditioning (IPC) (79, 80). In the first study in swine, two short IPC cycles (10 min LAD occlusion + 10 min reperfusion) were followed by reperfused MI (40 min LAD occlusion + 60 min reperfusion). After IPC, myocardial P-ERK levels increased. At the end of the protocol, the infarct size was smaller in animals subjected to IPC + MI compared with non-IPC MI controls. Notably, the local intramyocardial infusion of PD98059 or U0126 before IPC and during the IPC reperfusion period caused a reduction in P-ERK levels and blunted the protective IPC effects, leading to significantly larger infarct areas than those in the IPC + MI group (79). In a second similar study in rats, a sustained ischemia/reperfusion injury followed two short IPC cycles. This time, however, PD98059 was infused intramyocardially immediately after the long ischemia, during the initial 15 min of reperfusion. Again, PD98059 inhibited ERK phosphorylation and abrogated the IPC-induced protection, causing larger infarct regions comparable to those of the non-IPC + MI control group when assessed 2 h post-ischemia (80). In this last study, the Authors also investigated the effects of intramyocardial MEKI infusion in animals not subjected to IPC. When PD98059 was administered immediately after ischemia, during the reperfusion, it did not have any effect on the infarct scar, always measured 3 h post-ischemia (80).

These findings suggest that caution is needed when administering MEKI to MI patients, implying careful identification of the optimal therapeutic window to avoid interfering with the protective effects of physiological ERK activation after ischemia. However, the MEKI treatment effects in preclinical studies could be time- and model-dependent, and all the above studies present limitations. The last three studies in reperfused MI models assessed the MEKI effects after only a few hours post-MI. Instead, the first study showed the benefits of MEKI therapy 17 days post-MI but employed a permanent LAD occlusion model that neglects the revascularization procedure often applied to patients. For these reasons, additional studies in clinically relevant MI models and longer follow-ups are needed to extrapolate clinically meaningful information.

#### 6.1.2 Atherosclerosis and post-angioplasty remodeling

Modulating ERK signaling holds promise in slowing the progression of atherosclerosis and reducing the risk of cardiovascular events. According to a recent drug repurposing algorithm (OCTAD), PD0325901 was the top compound with predicted therapeutic benefits in atherosclerosis (175). In mice with advanced atherosclerotic lesions, U0126 contributed to blunting the atherogenic process and preserving the aorta wall's integrity by reducing macrophage and foam cell formation and lipids accumulation (168). We can expect that similar benefits are translated to other arteries. Other studies in mice suggested that MEKI could be therapeutic in atherosclerosis by modulating macrophage polarization toward the anti-inflammatory M2 phenotype (176).

MEKI treatment could also help prevent neointima formation and coronary artery restenosis after percutaneous coronary balloon angioplasty and stent placement, as suggested by studies on carotid arteries. In one study using rats, PD0185625 blocked carotid neointima formation 14 and 28 days after balloon injury (169). In another study conducted in a mouse model of neurofibromatosis type 1 with ligation-induced carotid artery injury, treatment with PD0325901 prevented the formation of carotid neointima when started before injury, while it reduced neointima formation when started after injury (170). The beneficial effects of MEKI in counteracting neointima formation can be explained by the protection from VSMC dedifferentiation towards the synthetic and proliferative phenotype and the attenuation of ERK-driven inflammatory response in ECs after stent fitting. PD0325901 also effectively prevented the osteoblast-like differentiation of VSMCs *in vitro*, thus suggesting it could exert a protective action against vascular calcification *in vivo* (97).

#### 6.1.3 Cardiac remodeling and heart failure

Preclinical studies suggest that MEKI could delay or revert pathological hypertrophy. In *in vitro* models of cardiac hypertrophy obtained by stimulating rat cardiomyocytes with phenylephrine or endothelin-1—two GPCR agonists -, Selumetinib, PD98059, PD184352, and U0126 inhibited ERK activation and attenuated markers of cardiac hypertrophy, like protein synthesis and cell size enlargement (172, 177, 178).

Transgenic mice expressing the activated form of the hepatocyte growth factor receptor—an RTK—develop concentric cardiac hypertrophy due to continuous activation of the MEK-ERK signaling. Pimasertib showed remarkable efficacy in attenuating hypertrophy in these mice, successfully preventing heart failure (179). Selumetinib attenuated pathological hypertrophy and heart remodeling in two rat models of pressure overload and ascending aortic constriction (172). In another study in rats, PD98059 attenuated cardiac hypertrophy induced by administering the NOS inhibitor L-NAME (173). MEKI drugs could also help manage HCM associated with Noonan syndrome. PD0325901 ameliorated cardiac function and corrected anatomical defects in a mouse model of Noonan syndrome (180).

MEKI are also expected to reduce cardiac fibroblast proliferation and ECM synthesis, thus attenuating cardiac fibrosis (126, 181).

#### 6.1.4 Aortic aneurysm

Several studies illustrated the efficacy of inhibiting the ERK signaling to delay aortic aneurysm progression using general compounds not directly targeting MEK. These clinically available agents include statins (182, 183), angiotensin-converting enzyme inhibitors (184), and calcium channel blockers (185). Despite the strong association between ERK activation and the pathological progression of aortic aneurysms, preclinical research in this field has been largely neglected.

In an *in-vitro* model of aortic aneurysm mimicked by treating aortic VSMCs with the monocyte chemoattractant protein-1*,* U0126 prevented the secretion of MMP9 (186). Likewise, U0126 restrained TNF-α-induced expression of MMP9 in aortic VSMCs (157). In another *in vitro* model of aortic aneurysm in which aortic VSMCs were exposed to oxidative stress to induce their switch from a contractile to synthetic phenotype, treatment with U0126 attenuated ERK activation and the cell phenotypic switch (143). These findings suggest that MEKI could counteract the MMP9 release and phenotypic switch in aortic VSMCs, two fundamental mechanisms responsible for aneurysm formation and progression.

Studies exploring the effects of MEKI in animal models of aortic aneurysms are still lacking. We found only one *in vivo* study in a mouse model of Marfan syndrome, in which the clinically available MEKI RDEA119 successfully prevented aortic root aneurysm formation (132). More research is urgently needed to confirm the promising therapeutic efficacy of MEKI in patients with aortic aneurysms.

### 6.2 Cardiovascular clinical studies with MEKI

Limited clinical studies have been conducted so far to assess the efficacy of MEKI in cardiovascular medicine. The data collected were, at times, incidental findings observed during clinical trials for cancer treatment.

Multiple case reports showed the successful off-label treatment with Trametinib in infants with Noonan Syndrome associated with severe early-onset eccentric hypertrophy and congestive heart failure (187–189). In all cases, treatment resulted in the complete remission or regression of cardiac hypertrophy. In some cases, it also improved the pulmonary valve stenosis. Trametinib also reversed heart failure secondary to hypertrophic cardiomyopathy in five patients with Costello Syndrome, a genetic disease caused by a germline gain-of-function mutation in the *HRAS* gene causing uncontrolled MEK activation (190). In all these studies, Trametinib was well tolerated with no serious adverse effects.

An ongoing phase II clinical trial (ClinicalTrials.gov ID NCT04258046) is investigating the therapeutic effects of Trametinib in children and adults with arteriovenous malformation, a progressive congenital vascular anomaly characterized by abnormal shunting of blood through dilated veins and arteries that causes complications including heart failure. A similar phase II clinical trial is set to start soon (ClinicalTrials.gov ID NCT06098872). Trametinib was already effective in reducing the size of the arteriovenous malformation and its blood flow in two patients (191, 192). Finally, Trametinib induced the complete resolution of vascular symptoms in a patient with Noonan Syndrome and severe lymphatic disorder (193).

## 7 Challenges for MEKI repurposing in cardiovascular patients

Despite the encouraging outcomes of the few preclinical and clinical cardiovascular studies with MEKI conducted so far, the clinical translation of this approach could be hampered by the potential MEKI toxicity and drug resistance.

### 7.1 Cardiovascular toxicity of MEKI

Clinically, MEKI drugs are well-tolerated and characterized by a high safety profile. A systematic review and meta-analysis of the cardiotoxicity of MEKI in cancer patients have revealed that the most significant adverse effects are increased risks of developing all-severity hypertension and asymptomatic reduced left ventricular ejection fraction (194). Side effects recorded in cancer patients under MEKI treatment include hypertension, heart failure and cardiac arrhythmias with incidence rates of 20%, 10% and 3%, respectively (195, 196). These off-target effects were of mild to moderate severity which were managed conservatively and resolved spontaneously upon course completion (197). Patients under 55 years old and who had a prolonged treatment duration of more than 6 months showed a higher incidence of side effects. Less than 1% of patients developed severe and refractory heart failure that obligated permanent drug discontinuation. Therefore, it has been recommended to do regular follow up with electrocardiography (ECG) monthly and echocardiography every 3 months (195).

At the cellular level, the underlying mechanism of hypertension, the most common side effect of MEKI, is decreased production of nitric oxide (NO) by ECs and peri-intimal VSMCs. Studies showed that MEK/ERK pathway inhibition is followed by a rebound over-activity phenomenon stimulating CD47 over-expression which in turn inhibits NO formation (198). Low NO impairs its vascular tone regulatory and anti-atherogenic effects and is associated with the development of hypertension (198). In cardiomyocytes, studies showed that inhibition of MEK/ERK pathway has pro-survival effects in physiological conditions and genetically predisposed cardiomyopathy patients (14, 199). However, on the other hand, inhibition of the anti-apoptotic factors’ transcription by MEK/ERK silencing stimulates stress-induced apoptosis in acute stressful conditions (199). Therefore, it has been concluded that MEK inhibition alone is not sufficient to induce cardiac adverse effects and must be accompanied by acute stressful factors such as ischemia or hypoxia (197).

### 7.2 MEKI resistance mechanisms

As described above, MEKI target MEK1/2 with high specificity and efficacy. However, as cancer studies have demonstrated, acquired MEKI resistance mechanisms are common and hamper therapeutic efficacy during long-term treatments. Indeed, MEK-ERK signaling inhibition can be easily bypassed by the activation of adaptive mechanisms that result in the same biological or transcriptional effects. These mechanisms include mutations of key targets of the MAPK pathway, the activation of upstream RTKs on the plasma membrane, and the activation of parallel signaling like the Phosphoinositide 3-kinase (PI3 K)/protein kinase B (AKT)/mammalian target of rapamycin (mTOR), Signal transducer and activator of transcription 3 (STAT3), and Hippo signaling pathways (200–202).

 Following MEKI treatment, melanoma cancer cells developed advantageous mutations of key molecules of the MAPK pathway—like RAS, RAF, and MEK—to achieve pathway hyperactivation (203–205). As described in Section 2, a physiological negative regulatory mechanism of the MAPK pathway is the suppression of the targets upstream of ERK1/2 by phosphorylated ERK, to avoid uncontrolled pathway activation. Consequently, in response to ERK repression by MEKI, upstream RTKs like EGFR, insulin-like growth factor receptor (IGF1R), platelet-derived growth factor receptor (PDGFR) and Erb-b2 receptor tyrosine kinase 3 (ERBB3) are reactivated (202). RTK reactivation leads to the initiation of the parallel PI3K/AKT signaling, which represents the primary mechanism of MEKI resistance in cancer cells and the leading mechanism driving cell proliferation and tumorigenesis in MEKI-treated cancer cells (206, 207). Activation of STAT3 (208) and Hippo (209) pathways are the other two mechanisms of adaptive resistance to MEKI. To overcome MEKI resistance, a solution explored in cancer patients is the combination of multiple drugs inhibiting the MAPK and parallel pathways simultaneously (200).

## 8 Final considerations and future directions

Targeting the RAS-RAF-MEK-ERK pathway in cardiovascular medicine represents a cutting-edge approach with the potential to transform how we diagnose and treat CVD. Here, we report some considerations about the advantages, challenges, and potential for future developments of MEKI therapeutics, including overcoming drug resistance and possible off-target and adverse effects.

### 8.1 One drug, many benefits

The MEK-ERK pathway has diverse roles within the cardiovascular system, influencing cardiomyocyte function, vascular tone, angiogenesis, inflammation, and more. Its multifaceted roles make it a versatile target in cardiovascular medicine. Because CVD is extraordinarily complex and often characterized by overlapping myocardial, vascular, metabolic, and immunologic alterations, a single drug targeting the MEK-ERK pathway could provide several benefits for the patient. For example, inhibiting the ERK pathway could help improve myocardial fibrosis, cardiomyocyte survival and contractility, cardiac function, vascular perfusion, and inflammation in the context of heart failure.

### 8.2 Precision medicine, diagnosis and target patient population

Precision medicine plays a pivotal role in overcoming resistance and reducing adverse effects. It is crucial to identify specific patient profiles and genetic markers associated with responsiveness to MEKI. By tailoring treatments to individual characteristics, clinicians can optimize therapeutic benefits while minimizing the risk of adverse effects. Identifying biomarkers and genetic testing can guide treatment decisions and help select the most appropriate patients for MEKI regimens. For example, as suggested by studies in transgenic mice, genetic alterations in MEK-ERK genes causing depressed ERK activity are associated with a worse prognosis after MI, implying that patients with these defects may not be amenable to MEKI therapy (57). MEKI therapy could not be suitable also for elderly patients presenting a natural decline in myocardial P-ERK levels and lacking the ability to activate ERK in response to an acute injury like MI (82). One remarkable advantage of precision medicine is its potential to enable early diagnosis and risk assessment. By identifying specific biomarkers or genetic variants associated with the MEK-ERK pathway, clinicians in the future could detect CVD at an earlier stage, often before symptoms become severe. This approach could lead to timely interventions, ultimately improving patient outcomes and reducing the overall burden of CVD.

### 8.3 Target disease, time, and duration of intervention

Determining the most suitable target disease and treatment regimen is also vital. It is important to remember that ERK signaling is central for the cells and organs to cope with acute stress, an element to consider when designing MEKI treatment protocols. In the MI context, for example, preclinical studies with MEKI using different MI models and timings of intervention provided apparent contrasting outcomes (64, 78–80). Therefore, additional studies are needed to reach clinically meaningful conclusions. Based on current experimental evidence, we can expect that MEKI therapy would better suit acute ischemic heart disease patients—in whom a short MEKI treatment could be offered to boost angiogenesis starting a few days after acute MI (64)—than patients with chronic ischemic heart disease, in whom a long-term MEKI treatment would not be suitable due to the insurgence of side effects seen in cancer patients. Preclinical and clinical studies suggested that individuals with cardiac hypertrophy could be another potential amenable target benefitting from MEKI treatment (172, 173, 179, 180, 187–189). Determining the best therapeutic window—both start and duration—for MEKI treatment will be crucial to maximizing benefits while reducing risks for patients. Given the limited current knowledge in the field, further studies are needed to explore the short- and long-term benefits and adverse effects of MEKI treatment and obtain information on the benefit/risk balance in CVD patients.

### 8.4 Dose optimization, combination therapies and patient surveillance

Finding the right balance between medication dosage and administration frequency is crucial to minimize side effects. Determining the lowest effective dose of MEKI is essential to achieve the desired therapeutic outcome, reducing the risk of side effects, including cardiac toxicity. Combining MEKI with other targeted agents or conventional treatments could enhance efficacy while reducing resistance. Combination therapies aim to maximize therapeutic benefits and overcome the limitations of single-agent treatments. By targeting multiple pathways simultaneously, clinicians can address the complexity of CVD. During treatment, rigorous patient monitoring and surveillance are vital to detect early signs of resistance or adverse effects. Regular assessment of cardiac function, biomarkers, and clinical symptoms can help healthcare providers adjust treatment regimens promptly and tailor them to individual patient needs.

### 8.5 Research and development of better drugs

Ongoing research efforts are focused on developing next-generation MEKI with high selectivity and reduced off-target effects. Continued research into the mechanisms of drug resistance and cardiotoxicity can inform the design of better-targeted medications. For example, the future development of a clinical-grade ERK-DI could allow direct targeting of the ERK kinase acting on the last part of the pathway selectively. By interfering with the ERK dimers formation, ERK-DI prevents the nuclear translocation of ERK1/2 and the resulting activation of the expression of genes responsible for cell cycle and growth, thus protecting against cardiac hypertrophy (28, 29, 52). Importantly, because ERK-DI does not block ERK phosphorylation/activation in the cell cytoplasm, the pro-survival activity of cytosolic ERK is safeguarded, a tremendous advantage to avoid cardiotoxicity in CVD patients (28, 29, 52).

## 9 Conclusions

The RAS-RAF-MEK-ERK signaling plays a central role in cardiovascular physiology and pathology. Although many preclinical studies encourage MEKI for CVD management, clinical studies are still lacking. If the balance between benefits and risks favors the first, and if the therapeutic effects of MEKI recorded in preclinical studies are confirmed in the clinical setting, in the future, targeted therapy with MEKI might represent a new frontier in CVD patient management.
